# Geraniin Ameliorates Haloperidol-Induced Orofacial Dyskinesia in Rats Through Mitigating Neuronal Oxidative Stress, Neuroinflammation, and Apoptosis via Modulation of the Nrf2 Signaling Pathway

**DOI:** 10.3390/ijms26125458

**Published:** 2025-06-06

**Authors:** Chih-Pei Hsu, Hsiang-Chien Tseng, Chih-Hsiang Fang, Yi-Wen Lin, Hung-Sheng Soung

**Affiliations:** 1 Division of Neurosurgery, Department of Surgery, Hsinchu Branch of Mackay Memorial Hospital, Hsinchu City 30071, Taiwan; 2 Department of Nursing, Mackay Junior College of Medicine, Nursing and Management, Taipei 11260, Taiwan; 3 Department of Anesthesiology, Shin Kong Wu Ho-Su Memorial Hospital, Taipei 11101, Taiwan; 4 School of Medicine, Fu Jen Catholic University, New Taipei City 24205, Taiwan; 5 Department of Orthopedics, College of Medicine, Taipei Medical University, Taipei 110, Taiwan; 6 Institute of Biomedical Engineering, National Taiwan University, Taipei 10051, Taiwan; 7 Department of Psychiatry, Yuan-Shan Branch of Taipei Veteran General Hospital, Yilan 26604, Taiwan; 8 Department of Biomedical Engineering, National Defense Medical Center, Taipei 11490, Taiwan

**Keywords:** geraniin, haloperidol, ML385, orofacial dyskinesia, striatum

## Abstract

Geraniin (GRN), an ellagitannin from Phyllanthus urinaria, shows antioxidant, anti-inflammatory, and neuroprotective effects. This study evaluated GRN’s potential against haloperidol (HPD)-induced orofacial dyskinesia (OD). Rats treated with HPD (1 mg/kg i.p.) for 21 days exhibited dopamine D2 receptor blockade, neurotoxicity, and OD, characterized by vacuous chewing movements (VCM) and tongue protrusion (TP). Then, 60 min post-HPD, GRN was administered i.p. daily for 21 days. OD behaviors were assessed, and on Day 21, striatal tissues were analyzed for oxidative stress, mitochondrial function, inflammation, and apoptosis. GRN alone did not cause OD but significantly reduced HPD-induced VCM and TP. It also reduced oxidative stress, improved antioxidant defense, preserved mitochondrial function, and decreased neuroinflammation and apoptosis. These effects were blocked by ML385, a nuclear factor erythroid-2-related factor 2 (Nrf2) pathway inhibitor. GRN protects against HPD-induced OD, likely via Nrf2 activation. It may be a promising candidate for TD therapy, pending further clinical investigation.

## 1. Introduction

Tardive dyskinesia (TD) is a drug-induced hyperkinetic movement disorder characterized by involuntary, repetitive motions predominantly involving the mouth, face, and tongue. These movements often take the form of choreiform, athetoid, or rhythmic patterns. TD is widely acknowledged as a delayed adverse reaction to prolonged neuroleptic therapy, particularly with first-generation antipsychotics. Despite discontinuation of the offending agent, symptoms may persist, with an estimated 2% of cases becoming irreversible, posing a significant therapeutic challenge in managing schizophrenia [1,2,3]. Haloperidol (HPD), a commonly used first-generation butyrophenone-class antipsychotic, is effective in managing acute agitation in schizophrenia but, when administered over 21 consecutive days, strongly blocks dopamine D2 receptors. This blockade induces neurotoxicity and can lead to orofacial dyskinesia (OD), characterized by increased vacuous chewing movements (VCM), tongue protrusion (TP), orofacial bursts, and cataleptic behavior in rodent models—phenotypes that closely resemble clinical TD. These models have long served as reliable tools to study the pharmacological and neuropathological underpinnings of TD [4]. Research indicates that HPD-induced OD is associated with increased striatal nitric oxide (NO) production, elevated lipid peroxidation (LPO) byproducts, heightened proinflammatory cytokines, reduced antioxidant enzyme activity, and significant neurodegeneration in the striatum, all contributing to TD-like motor deficits in rats [5,6,7,8,9,10]. Although the exact pathophysiological mechanisms of OD remain to be fully elucidated, prior studies suggest that antioxidant or anti-inflammatory therapies may alleviate nitrosative and oxidative stress, neuroinflammation, and neuronal damage in the striatum, offering a promising therapeutic strategy for TD in preclinical models [5,6,7,8,9,10,11].

Current pharmacological treatments for TD often show limited efficacy and are frequently accompanied by adverse effects, highlighting the urgent need for novel therapeutic strategies [1,2,3]. Emerging preclinical evidence suggests that geraniin (GRN), the primary polyphenolic compound in *Nephelium lappaceum* L., exhibits a broad range of pharmacological activities, including antiviral, antitumor, anti-inflammatory, and antihypertensive effects [12,13,14,15,16]. Notably, GRN is a potent antioxidant that scavenges reactive radicals, boosts endogenous antioxidant defenses, reduces lipid peroxidation, and preserves mitochondrial catalase activity—effects likely mediated through the Nrf2 signaling pathway [15,17,18,19,20]. GRN has shown neuroprotective efficacy in models of ischemia/reperfusion injury, nephrotoxicity, and toxin-induced cognitive impairment. However, despite its diverse pharmacological benefits, the neuroprotective potential of GRN against HPD-induced OD as well as the mechanisms underlying these effects remain unexplored. Based on its well-documented antioxidative, anti-inflammatory, anti-apoptotic, and neuroprotective activities, we hypothesize that GRN could prevent OD development [12,15,16,17,18,19,20].

This research aimed to evaluate the neuroprotective potential of GRN against OD induced by HPD, with a particular focus on the involvement of the Nrf2-dependent signaling pathway. To achieve this goal, several key parameters were investigated: (1) Behavioral Assessment: The study assessed the severity of OD by measuring VCM and TP, two hallmark behavioral indicators commonly associated with orofacial dyskinesia. (2) Oxidative and Nitrosative Stress Markers: Levels of nitric oxide (NO) and malondialdehyde (MDA) were quantified to evaluate oxidative and nitrosative damage, both of which are closely linked to HPD-induced neurotoxicity. (3) Endogenous Antioxidant Defense: The concentrations of crucial antioxidant enzymes—glutathione (GSH), superoxide dismutase (SOD), and catalase (CAT)—were analyzed to determine GRN’s effect on restoring redox homeostasis. (4) Mitochondrial Function: Mitochondrial bioenergetics were assessed through multiple enzymatic activities, including succinate dehydrogenase (SDH), total ATPase, NADH–cytochrome C reductase (complexes I–III), and succinate–cytochrome C reductase (complexes II–III), to understand GRN’s role in preserving mitochondrial integrity. (5) Neuroinflammation: The expression levels of proinflammatory cytokines—tumor necrosis factor-alpha (TNF-α), interleukin-1 beta (IL-1β), and interleukin-6 (IL-6)—were measured to assess GRN’s anti-inflammatory potential in the striatum. (6) Apoptosis Evaluation: Caspase-3 activity was used as a marker to gauge the extent of apoptosis, particularly in the striatal region affected by HPD treatment.

These investigative parameters were selected based on existing literature, which links HPD-induced OD with striatal oxidative/nitrosative stress, mitochondrial impairment, neuroinflammatory responses, and progressive neurodegeneration [5,6,7,8,9,10,11,21,22]. To clarify the contribution of the Nrf2 pathway in mediating GRN’s effects, a specific inhibitor of nuclear factor erythroid 2-related factor 2 (Nrf2), ML385, was co-administered with GRN in a subset of experimental groups. This allowed for a more detailed understanding of whether GRN’s protective actions were dependent on Nrf2 activation.

## 2. Results

Prior to HPD administration, the measured parameters showed comparable values across all groups. According to Tukey’s post hoc analysis, there were no statistically significant differences between the GRN-treated groups (1, 3, 10, 30, or 100 mg/kg; G1, G3, G10, G30, or G100) and the control group (C), as indicated by non-significant *p*-values (G1 vs. C, *p* > 0.05; G3 vs. C, *p* > 0.05; G10 vs. C, *p* > 0.05; G30 vs. C, *p* > 0.05; and G100 vs. C, *p* > 0.05).

### 2.1. Effect of HPD and GRN on OD Parameters (VCM and TP)

VCM and TP were used as behavioral indicators to evaluate HPD-induced orofacial dyskinesia. Compared to the control group (C), HPD treatment led to a significant, time-dependent increase in the frequency of VCM and TP (Table 1 and Table 2) on Days 1, 7, 14, and 21 (Day 1: VCM increased by 15.54-fold, TP by 3.35-fold, H vs. C, *p* < 0.001; Day 7: VCM by 18.49-fold, TP by 5.1-fold, H vs. C, *p* < 0.001; Day 14: VCM by 26.57-fold, TP by 10.69-fold, H vs. C, *p* < 0.001; and Day 21: VCM by 50.63-fold, TP by 19.6-fold, H vs. C, *p* < 0.001). Treatment with GRN at doses of 1 and 3 mg/kg showed no significant effect on the HPD-induced increase in VCM and TP across all time points (H + G1 vs. H, *p* > 0.05; and H + G3 vs. H, *p* > 0.05). Similarly, GRN at 10, 30, and 100 mg/kg did not significantly alter VCM or TP frequencies on Days 1 and 7 (H + G10 vs. H, *p* > 0.05; H + G30 vs. H, *p* > 0.05; and H + G100 vs. H, *p* > 0.05). However, by Day 21, GRN at 10 mg/kg significantly reduced VCM and TP compared to the HPD group (VCM: −34.16%, TP: −31.65%, H + G10 vs. H, *p* < 0.001). GRN at 30 and 100 mg/kg significantly reduced both parameters on Day 14 (G30: VCM −25.41%, TP −38.29%; G100: VCM −32.44%, TP −53.4%; all *p* < 0.001) and Day 21 (G30: VCM −50.24%, TP −50.27%; G100: VCM −57.38%, TP −59.81%; all *p* < 0.001). Based on these behavioral outcomes, GRN at 10 and 30 mg/kg was chosen as the optimal dosing for the study, as the effects of 30 mg/kg were comparable to those of 100 mg/kg. Furthermore, the co-administration of ML385, an Nrf2 pathway inhibitor, nearly abolished the protective effects of GRN at both doses (H + M + G10 vs. H, *p* > 0.05; H + M + G30 vs. H, *p* > 0.05), indicating that the neuroprotective effects of GRN against HPD-induced orofacial dyskinesia are mediated via the Nrf2 signaling pathway.

### 2.2. The Effect of HPD and GRN on the Nitrite and MDA Levels in the Striatum

**Figure 1:**
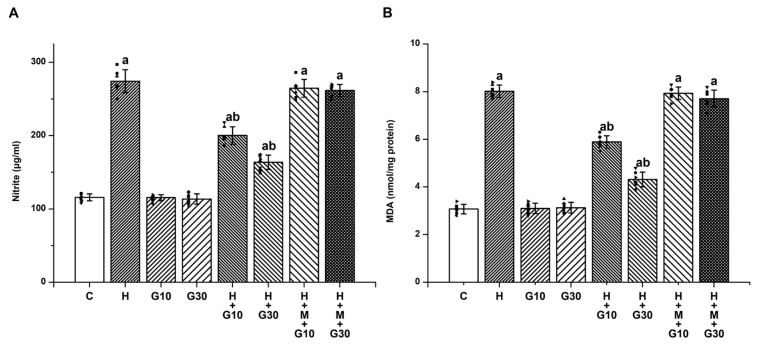
To evaluate GRN’s modulatory effect on HPD-induced nitrosative and oxidative stress in the rat striatum, the concentrations of (**A**) nitrite and (**B**) MDA were quantified. Results are reported as mean ± SEM (*n* = 8). Statistical comparisons were performed using one-way ANOVA followed by Tukey’s post hoc test. Statistical significance was defined as “a” *p* < 0.001 relative to the control group (C); “b” *p* < 0.001 relative to the HAL-treated group (H). Squares, triangles and circles are individual data points.

Analysis of rat striatal tissue revealed that treatment with HPD significantly elevated markers of oxidative and nitrosative stress. Nitrite levels rose from 115.71 ± 4.68 to 274.14 ± 15.55 μg/mL (Figure 1A) and MDA from 3.07 ± 0.2 to 8.01 ± 0.26 nmol/mg protein (Figure 1B) by Day 21 (both *p* < 0.001), indicating substantial free radical formation. Administration of GRN at 10 mg/kg and 30 mg/kg effectively mitigated these increases in a dose-dependent manner. Specifically, nitrite levels dropped by 46.71% and 69.76%, while MDA levels fell by 42.91% and 74.9%, respectively (*p* < 0.001 for all comparisons). Co-treatment with the Nrf2 inhibitor ML385, however, abolished these protective effects, highlighting the pivotal role of the Nrf2 pathway.

### 2.3. The Effect of HPD and GRN on the GSH, SOD, and CAT Levels in the Striatum

**Figure 2:**
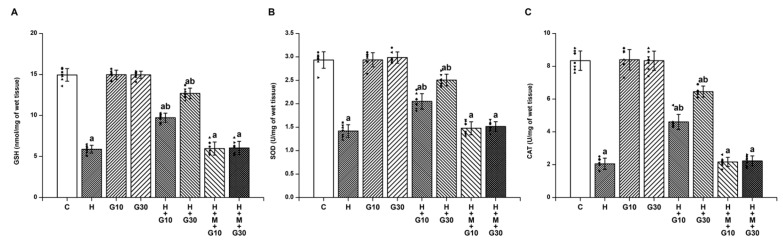
The potential of GRN to counteract HPD-induced depletion of endogenous antioxidants was assessed by measuring striatal levels of (**A**) GSH, (**B**) SOD, and (**C**) CAT. Results are reported as mean ± SEM (*n* = 8). Significance was considered at “a” *p* < 0.001 relative to the control group (C); “b” *p* < 0.001 relative to the HAL group (H). Squares, triangles and circles are individual data points.

In contrast, antioxidant defenses were markedly suppressed in the H group. On Day 21, GSH levels fell from 14.94 ± 0.77 to 5.89 ± 0.48 nmol/mg tissue (Figure 2A), SOD from 2.93 ± 0.18 to 1.42 ± 0.13 U/mg tissue (Figure 2B), and CAT from 8.34 ± 0.59 to 2.06 ± 0.35 U/mg tissue (all *p* < 0.001) (Figure 2C). GRN administration restored these levels significantly: at 10 mg/kg, GSH increased by 42.43%, SOD by 41.72%, and CAT by 40.54%; at 30 mg/kg, GSH rose by 75.14%, SOD by 72.19%, and CAT by 70.11% (*p* < 0.001 for all). The beneficial effects of GRN were again reversed by ML385 co-treatment, reinforcing the involvement of Nrf2 signaling in GRN-mediated antioxidative defense.

### 2.4. The Effect of HPD and GRN on the Mitochondrial Enzyme Activities in the Striatum

**Figure 3:**
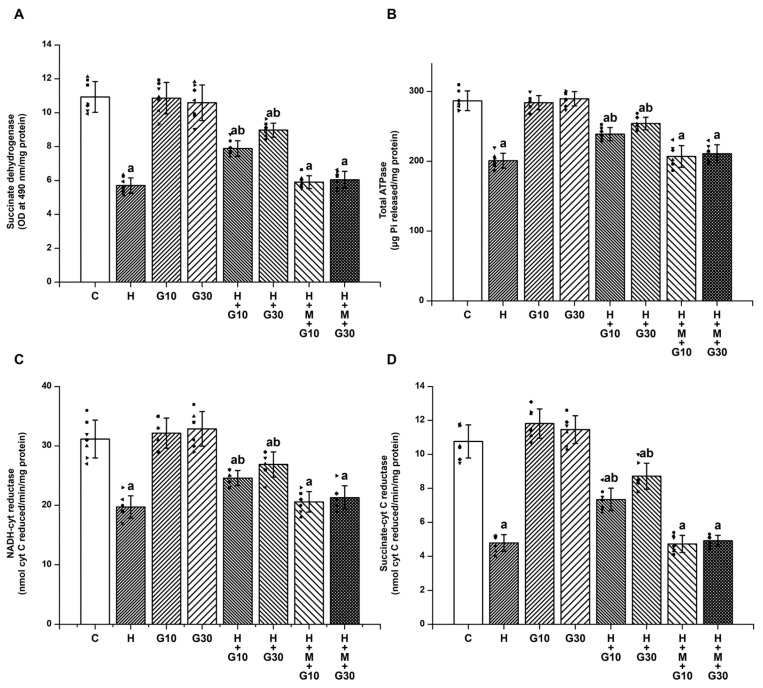
To determine GRN’s impact on HPD-related mitochondrial dysfunction, enzymatic activities were evaluated for (**A**) SDH, (**B**) total ATPase, (**C**) NADH–cytochrome C reductase (complexes I–III), and (**D**) succinate–cytochrome C reductase (complexes II–III). All values are shown as mean ± SEM (*n* = 8). Statistical significance was determined by one-way ANOVA followed by Tukey’s post hoc test, with “a” *p* < 0.001 relative to control (C); “b” *p* < 0.001 relative to HAL-treated (H). Squares, triangles and circles are individual data points.

The striatal levels of SDH (Figure 3A), total ATPase (Figure 3B), NADH-cytochrome C reductase (Figure 3C), and succinate-cytochrome C reductase (Figure 3D) were significantly reduced in the H group (SDH: from 10.93 ± 0.91 to 5.7 ± 0.45 OD at 490 nm/mg protein, H vs. C, *p* < 0.001; total ATPase: from 286.57 ± 14.12 to 200.68 ± 10.73 μg Pi released/mg protein, H vs. C, *p* < 0.001; NADH-cytochrome C reductase: from 31.14 ± 3.18 to 19.71 ± 1.89 nmol cyt C reduced/min/mg protein, H vs. C, *p* < 0.001; and succinate-cytochrome C reductase: from 10.76 ± 0.98 to 4.79 ± 0.49 nmol cyt C reduced/min/mg protein, H vs. C, *p* < 0.001) on Day 21. Similarly, HPD exposure led to marked impairments in mitochondrial enzyme activity. GRN at both tested doses significantly restored the function of SDH, ATPase, and complexes I–III and II–III. At 10 mg/kg, values were SDH: 7.89 ± 0.46 OD/mg protein; ATPase: 238.86 ± 9.44 μg Pi/mg protein; NADH-cytochrome C reductase: 24.57 ± 1.27 nmol/min/mg; and succinate-cytochrome C reductase: 7.34 ± 0.66 nmol/min/mg. At 30 mg/kg, the corresponding values were 8.97 ± 0.41, 254.14 ± 8.97, 26.86 ± 2.12, and 8.71 ± 0.76, respectively (*p* < 0.001 for all comparisons vs. H group). These mitochondrial benefits were significantly diminished when GRN was co-administered with ML385, once again pointing to the essential role of Nrf2 in regulating mitochondrial integrity under neurotoxic conditions.

### 2.5. The Effect of HPD and GRN on the TNF-α, IL-1β, IL-6, and Caspase-3 Levels in the Striatum

**Figure 4:**
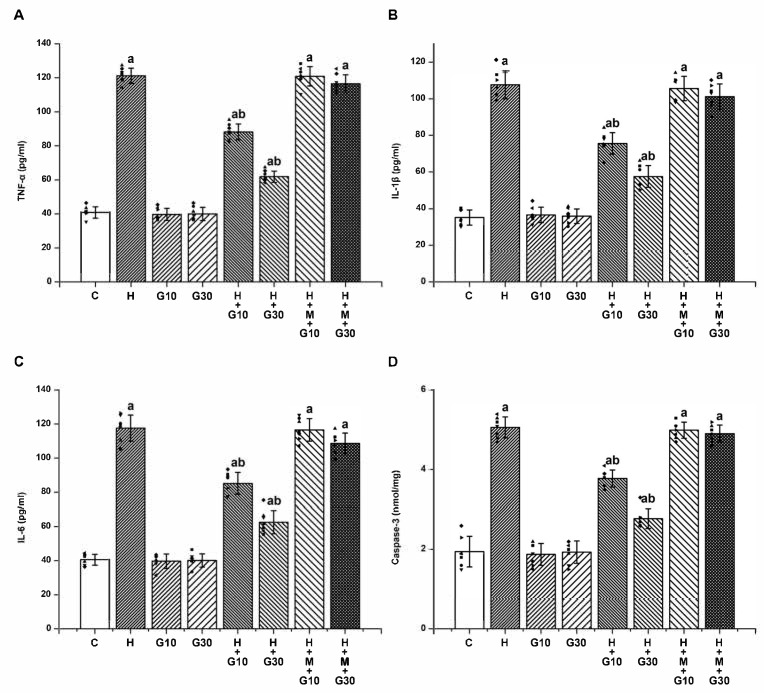
The effects of GRN on HPD-induced neuroinflammation and apoptosis in the striatum were assessed by quantifying (**A**) TNF-α, (**B**) IL-1β, (**C**) IL-6, (**D**) caspase-3 activity. Results are reported as mean ± SEM (*n* = 8). Statistical evaluation was carried out using one-way ANOVA followed by Tukey’s post hoc analysis. Significance levels were set at “a” *p* < 0.001 relative to the control group (C); “b” *p* < 0.001 relative to the HAL group (H). Squares, triangles and circles are individual data points.

Furthermore, 21-day HPD treatment led to significant elevations in proinflammatory cytokines and apoptotic signaling in the striatum. TNF-α increased from 40.86 ± 3.34 to 121.14 ± 4.45 pg/mL (Figure 4A), IL-1β from 35.14 ± 4.14 to 107.57 ± 7.68 pg/mL (Figure 4B), IL-6 from 40.57 ± 3.21 to 117.57 ± 7.63 pg/mL (Figure 4C), and caspase-3 from 1.94 ± 0.39 to 5.06 ± 0.26 nmol/mg protein (*p* < 0.001 for all) (Figure 4D). GRN significantly attenuated these elevations in a dose-dependent manner. At 10 mg/kg, cytokine levels were reduced to TNF-α: 88.14 ± 4.67; IL-1β: 75.57 ± 5.86; IL-6: 85.14 ± 6.47 pg/mL; and caspase-3: 3.77 ± 0.21 nmol/mg. At 30 mg/kg, reductions were even more pronounced: TNF-α: 61.86 ± 3.29; IL-1β: 57.57 ± 5.94; IL-6: 62.57 ± 6.78 pg/mL; and caspase-3: 2.77 ± 0.25 nmol/mg protein (*p* < 0.001). Co-treatment with ML385 nullified GRN’s anti-inflammatory and anti-apoptotic actions, confirming the Nrf2 pathway’s central role in mediating GRN’s neuroprotective effects.

## 3. Discussion

Through a combination of behavioral assessments and biochemical analyses in animal models, this study provides strong evidence that GRN offers notable protection against HPD-induced OD in rats. Prolonged administration of HPD (1 mg/kg, i.p., for 21 consecutive days) was shown to induce neurotoxic effects, manifesting in clear signs of OD. These behavioral alterations were closely associated with increased oxidative and nitrosative stress, mitochondrial impairment, neuroinflammatory responses, and apoptotic activity within the striatum. Importantly, GRN, when administered alone, did not elicit any dyskinetic behavior but significantly counteracted HPD-induced OD, as indicated by reductions in VCM and TP. The compound exerted its protective effect by alleviating oxidative and nitrosative stress, improving antioxidant defenses, preventing mitochondrial impairment, and suppressing inflammation and apoptosis in the striatal region. Notably, the neuroprotective mechanism of GRN was shown to involve the Nrf2 signaling pathway. This was evidenced by the use of ML385, a known Nrf2 inhibitor, which effectively negated the beneficial actions of GRN. To our knowledge, this study is the first to demonstrate GRN’s robust capacity to protect against HPD-induced neurodegenerative changes, underscoring its promise as a therapeutic candidate for managing drug-induced movement disorders in future clinical applications.

The HPD-induced model, established through daily intraperitoneal administration of 1 mg/kg for 21 days, is a well-validated and widely used approach for studying TD. Among existing animal models, this paradigm is considered highly relevant due to its consistent efficacy and strong resemblance to clinical TD. In particular, the orofacial motor disturbances elicited by HPD closely mimic the purposeless oral movements characteristic of TD in humans. Furthermore, HPD has been directly implicated in the clinical manifestation of TD, supporting its translational value in preclinical research. The induction of VCM and TP in rats serves as a reliable behavioral indicator of dyskinesia, and has become a valuable tool for screening and evaluating potential therapeutic interventions for TD [4,6,10]. Despite ongoing discussions regarding the limitations of animal models for TD, the HPD-induced OD model continues to offer useful insights. Our current findings—consistent with prior studies using the same model [6,7,8,9,10,11,21]—confirm the successful induction of OD following 21 days of HPD treatment. This was evidenced by a marked increase in VCM and TP frequency. Moreover, HPD exposure led to impairments in oxidative defense mechanisms, mitochondrial impairment, and degeneration within the striatum, collectively indicating a neurotoxic profile that parallels TD-like pathology. Recognizing the potential influence of sex-based pharmacological variability, this study utilized equal numbers of male and female animals. Nonetheless, further investigations are warranted to fully unravel the sex-specificity and molecular mechanisms underlying HPD-induced OD.

HPD-induced OD has been closely linked to elevated nitrosative and oxidative stress in the striatum, alongside neuroinflammatory responses—factors collectively contributing to progressive neuronal injury [5,6,7,8,9,10,11,21,23,24]. In line with this, our biochemical analyses demonstrated that HPD administration significantly increased striatal nitrite and MDA levels, markers of nitrosative and lipid peroxidation-related damage, respectively. Concurrently, GSH, a key endogenous antioxidant, were markedly reduced, and the enzymatic activities of SOD and CAT—key antioxidant components—were markedly decreased. Furthermore, HPD treatment impaired mitochondrial function, as indicated by disruptions in SDH and ATPase activities, as well as enzymes of the electron transport chain, highlighting the pivotal role of reactive oxygen and nitrogen species (ROS/RNS) and mitochondrial impairment in HPD-induced neurotoxicity. Nevertheless, further studies are needed to directly quantify ROS/RNS levels. As a first-generation butyrophenone-class antipsychotic, HPD exerts its therapeutic effect by antagonizing dopamine D2 receptors, which consequently elevates dopamine (DA) turnover. This heightened DA metabolism facilitates the formation of neurotoxic metabolites, including hydrogen peroxide, thereby exacerbating oxidative stress in dopaminergic neurons [6,9,10]. Additionally, DA undergoes autoxidation to produce o-quinone aminochrome, which is subsequently reduced to leukoaminochrome o-semiquinone radicals—potent sources of endogenous free radicals. The combination of increased DA turnover and HPD-induced enhancement of glutamatergic transmission contributes to excessive free radical production, promoting oxidative injury, neuronal degeneration, and ultimately, the development of OD [11,24,25].

HPD has also been shown to impair mitochondrial integrity by inhibiting complex I (NADH: ubiquinone oxidoreductase) of the electron transport chain, thereby impairing respiration and elevating ROS production [25,26,27,28,29]. This mitochondrial impairment results in diminished ATP production and suppression of Na⁺/K⁺-ATPase activity, contributing to progressive neuronal depolarization. Such depolarization alleviates the voltage-dependent magnesium (Mg^2^⁺) blockade of N-methyl-D-aspartate (NMDA) receptors, rendering them more susceptible to calcium (Ca^2^⁺) influx and subsequent excitotoxicity [30]. The ensuing overactivation of NMDA receptors facilitates excessive Ca^2^⁺ entry into neurons, which not only amplifies ROS and RNS production but also promotes lipid peroxidation, mitochondrial degradation, and nuclear DNA fragmentation [30,31]. These deleterious effects are further compounded by a bidirectional interplay between mitochondrial dysfunction and NMDA receptor overactivation, creating a vicious cycle of escalating oxidative and nitrosative stress in the presence of HPD. In addition, NO has been shown to inhibit enzymes crucial for cellular energy metabolism, thereby worsening oxidative injury, particularly under pathological conditions characterized by heightened ROS/RNS levels [10,26,29,32]. Taken together, these findings provide compelling evidence that mitochondrial impairment and redox imbalance are central to the pathophysiology of HPD-induced OD.

Excessive nitrosative and oxidative stress also triggers inflammatory signaling cascades, culminating in the release of proinflammatory cytokines such as TNF-α, IL-1β, and IL-6. These mediators are critically involved in triggering apoptotic pathways and have been implicated in neuronal cell death and the acceleration of OD progression [7,21,23,33]. In line with earlier research, our study found significantly elevated levels of these cytokines in the striatum of HPD-treated animals, reinforcing the role of neuroinflammation in HPD-induced striatal damage, which are key contributors to the manifestation of OD. 

Furthermore, caspase-3, a central executioner in the apoptotic cascade, is known to mediate both biochemical and structural features of programmed neuronal death [26,29,32,34,35]. The marked upregulation of caspase-3 expression observed in HPD-treated animals in this study provides additional evidence for the involvement of apoptotic processes in the pathogenesis of striatal degeneration and behavioral abnormalities associated with OD. These findings are consistent with previous experimental reports showing that chronic HPD exposure disrupts normal neuronal function by inducing neuronal damage or loss, thereby promoting OD-like symptoms in animal models [21,33,36].

In clinical settings, TD is often managed using conventional antipsychotic medications in combination with anticholinergic agents such as biperiden. However, anticholinergic drugs often produce undesirable side effects—including tachycardia, mydriasis, xerostomia, and urinary retention—and may exacerbate the positive symptoms of schizophrenia [1,2,3]. These drawbacks underscore the pressing need for novel and safer therapeutic strategies for TD. GRN, a major polyphenolic compound derived from *Nephelium lappaceum* L., has garnered attention due to its potent antioxidant, anti-inflammatory, and anti-apoptotic properties, along with its ability to mitigate neurochemical imbalances [15,16,17,18,19,20]. In the present study, GRN demonstrated significant neuroprotective effects against HPD-induced OD in rats. GRN treatment notably mitigated the HPD-induced elevations in nitrite, MDA, proinflammatory cytokines (TNF-α, IL-1β, IL-6), and caspase-3 activity within the striatum. Simultaneously, it restored mitochondrial function and antioxidant defenses, as evidenced by increased activities of SDH, total ATPase, ETC) complexes, as well as elevated levels of GSH, SOD, and CAT. Conversely, when GRN was co-administered with ML385—a selective inhibitor of the Nrf2 signaling pathway—the beneficial biochemical effects were significantly diminished. This reversal strongly suggests that the neuroprotective actions of GRN are at least partially dependent on the activation of the Nrf2-mediated cellular defense mechanism.

The antioxidant capacity of GRN is largely attributed to its capacity to directly scavenge ROS and preserve the functionality of antioxidant enzymes [16,17,18,20]. GRN acts as an efficient radical scavenger by donating electrons and has been shown to upregulate Nrf2 expression, thereby enhancing the activity of essential antioxidant enzymes including GSH, SOD, and CAT. In addition to its antioxidant effects, GRN is hypothesized to exert anti-inflammatory activity through inhibition of the nuclear factor kappa-light-chain-enhancer of activated B cells (NF-κB) signaling pathway, resulting in the downregulation of proinflammatory cytokines such as TNF-α, IL-1β, and IL-6 [12,15,16,17,19]. Moreover, GRN has been shown to activate several pro-survivals signaling pathways, including PI3K/Akt, ERK1/2, and protein kinase C (PKC), which contribute to the upregulation of anti-apoptotic proteins such as Bcl-2 and Nrf2, while downregulating pro-apoptotic mediators [16,18,20]. These mechanisms collectively confer robust neuroprotective effects against HPD-induced oxidative stress, neuroinflammation, and apoptosis, thereby supporting neuronal survival and functional integrity. Importantly, GRN administration alone did not induce OD, indicating that its protective effects do not interfere with normal motor function but rather target pathological changes induced by HPD. These findings highlight the promising therapeutic potential of polyphenolic compounds like GRN in the clinical management of TD.

## 4. Materials and Methods

### 4.1. Animals

All experimental procedures were conducted in strict accordance with the “Guidelines for the Care and Use of Laboratory Animals” issued by the U.S. National Institutes of Health. Prior to the start of the study, the protocol had received approval from the Institutional Animal Care and Use Committee (IACUC) of the National Taiwan University College of Medicine (Approval No: 20210729). The subjects of the study were Wistar rats, approximately three months old and weighing between 270 and 300 g, which were procured from BioLASCO Co., Ltd. (Taipei, Taiwan). The animals were housed in groups of three per Plexiglas cage under standardized conditions: a 12-h light/dark cycle (lights on at 7:00 a.m.), temperature maintained at 22 ± 3 °C, with ad libitum access to food and water. To reduce stress and ensure the animals’ well-being, each rat underwent gentle handling for 20 min daily over a period of 7 days before the commencement of any experimental procedures.

### 4.2. Drugs

Haloperidol (HPD; CAS No. 52-86-8, product code H1512-10G) and ML385 (CAS No. 846557-71-9, product code SML1833-25MG) were sourced from Sigma-Aldrich (St. Louis, MO, USA) and prepared in sterile normal saline. Geraniin (GRN; purity ≥95%, CAS No. 60976-49-0, product code PHL80994), likewise obtained from Sigma-Aldrich, was first dissolved in 1% dimethyl sulfoxide (DMSO) to ensure solubility, and subsequently diluted to the appropriate working concentrations with normal saline. Each drug solution was freshly formulated immediately before administration to maintain chemical stability and biological activity. The dosing strategies for all agents adhered to previously validated experimental protocols described in published studies [17,26,37]. Intraperitoneal (i.p.) injections were administered once daily for a period of 21 consecutive days, with a standardized injection volume of 2.0 mL per kilogram of body weight.

### 4.3. Experimental Groups and Treatment Protocols

For the preliminary phase, rats were assigned randomly to twelve groups (n = 8 per group; balanced for sex), as follows: 

I. Control group (C): normal saline (i.p.) for 21 days;

II. HPD treatment group (H): HPD (1 mg/kg i.p.) for 21 days;

III–VII. GRN 1 or 3 or 10 or 30 or 100 mg/kg treatment group (G1, G3, G10, G30, G100): GRN (1 or 3 or 10 or 30 or 100 mg/kg, i.p.) for 21 days;

VIII–XII. HPD + GRN 1 or 3 or 10 or 30 or 100 mg/kg treatment group (H + G1, H + G3, H + G10, H + G30, H + G100): HPD + GRN (1 or 3 or 10 or 30 or 100 mg/kg, i.p.) for 21 days.

Preliminary behavioral evaluations demonstrated that GRN doses of 10, 30, and 100 mg/kg significantly attenuated HPD-induced OD, as measured by vacuous chewing movements VCM and TP. Since GRN at 30 and 100 mg/kg yielded comparable results, doses of 10 and 30 mg/kg were selected for further investigation. To elucidate the involvement of the Nrf2 signaling pathway, two additional groups were added:

XIII, XIV. HPD + ML385 + GRN 10 or 30 mg/kg treatment group (H + M + G10, H + M + G30): HPD + ML385 (30 mg/kg; i.p.) + GRN (10 or 30 mg/kg, i.p.) for 21 days.

**Figure 5:**
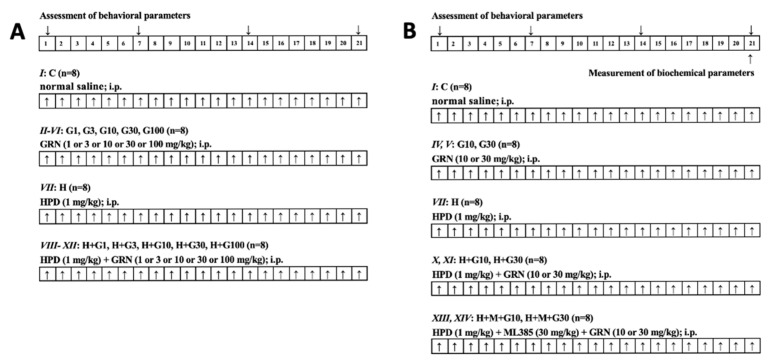
Experimental design and drug treatment paradigm of (**A**) preliminary study on behavioral assessment and (**B**) further study on behavioral and biochemical measurement. ↑: Drugs treatment.

In these groups, ML385 was administered 30 min prior to GRN, which was administered 60 min after HPD. Following the completion of behavioral testing, rats from Groups I, IV, V, VII, X, XI, XIII, and XIV underwent further biochemical analyses targeting the striatum to investigate the underlying mechanisms of GRN’s effects in greater detail. A schematic overview of the experimental protocol is provided in Figure 5.

### 4.4. Behavioral Assessment of OD

OD was assessed on Days 1, 7, 14, and 21, approximately 6 h post-injection, following established laboratory protocols. To eliminate observer bias, animals were assigned random numeric codes. Two independent, blinded observers conducted all behavioral assessments. During testing, rats were placed individually in a transparent observation chamber (20 cm × 20 cm × 19 cm), equipped with mirrored flooring to enable full visualization of facial movements, even when it was facing away from the observer. After a 2-min acclimation period, OD-related behaviors—including VCM and TP—were recorded for a duration of 5 min. All behavioral evaluations were carried out consistently between 9:00 a.m. and 11:00 a.m. to control for circadian influences.

### 4.5. Biochemical Measurement

On the final experimental day (Day 21), rats from designated groups (I, IV, V, VII, X, XI, XIII, and XIV) were sacrificed one hour after the last behavioral evaluation to enable biochemical analysis. Brains were rapidly excised, briefly rinsed in ice-cold isotonic saline to eliminate residual blood, and promptly stored at −80 °C to preserve tissue integrity. The striatum was meticulously isolated on a chilled dissection platform, using anatomical references adapted from Budantsev et al. [38]. Dissected striatal tissues were gently washed in physiological saline, accurately weighed, and homogenized in 0.1 N hydrochloric acid. For downstream analyses, a 10% (*w*/*v*) homogenate was prepared in 0.1 M phosphate buffer (pH 7.4), serving as the base for various biochemical assays. To evaluate catalase (CAT) activity, homogenates were centrifuged at 1000× *g* for 20 min at 4 °C to obtain the post-nuclear supernatant. For the measurement of additional enzymatic activities, such as those related to mitochondrial or antioxidant function, a second centrifugation was conducted at 12,000× *g* for 60 min at 4 °C, yielding the supernatant fractions required for further analysis.

### 4.6. Nitrites Concentration

Nitrite levels, serving as an indirect measure of nitric oxide (NO) production, were determined by the Roche NO Colorimetric Assay Kit (Basel, Switzerland). This method is based on a diazotization reaction wherein nitrite reacts with sulfanilamide and subsequently couples with N-(1-naphthyl) ethylenediamine dihydrochloride, forming a stable azo chromophore detectable at a wavelength of 540 nm. For sample preparation, 100 μL of striatal tissue homogenate was mixed with 400 μL of redistilled water and incubated in a boiling water bath for 15 min to denature endogenous enzymes. Once cooled to room temperature, 30 μL each of Carrez I (0.36 M potassium hexacyanoferrate (II) trihydrate) and Carrez II (1 M zinc sulfate heptahydrate) were added sequentially to precipitate proteins. The mixture was adjusted to pH 8.0 by the addition of 4 μL of 10 M sodium hydroxide, followed by centrifugation at 10,000× *g* to obtain a clear supernatant. A 75 μL aliquot of the resulting supernatant was transferred to a microplate well and combined with 75 μL of redistilled water. For blank controls, water was substituted for the sample. After incubation at 25 °C for 30 min, 50 μL of 1% sulfanilamide solution (prepared in 2.5% phosphoric acid) and 50 μL of 0.1% N-(1-naphthyl) ethylenediamine dihydrochloride solution (also in 2.5% phosphoric acid) were added. The plate was protected from light and incubated for 15 min to ensure complete color development. Absorbance was subsequently recorded at 540 nm. Nitrite concentrations were extrapolated from a standard calibration curve generated using sodium nitrite (range: 6–600 μM) and expressed in micrograms per milliliter (μg/mL), representing relative nitric oxide content in the striatal tissue.

### 4.7. MDA 

To evaluate lipid peroxidation, MDA levels in striatal tissue were determined using the method of Wills et al. [39]. Equal volumes of striatal homogenate and Tris–HCl buffer were incubated at ambient temperature for 2 h. The mixture was then treated with 10% trichloroacetic acid to precipitate proteins, followed by centrifugation to collect the supernatant. Thiobarbituric acid (TBA) was added to this supernatant, and the solution was heated in a water bath for 10 min to promote the formation of the MDA–TBA adduct. After cooling, the resulting chromophore was quantified by measuring absorbance at 532 nm using a BioTek Microplate Reader (Model BTFLX800TB; Agilent Technologies, Santa Clara, CA, USA). MDA levels were calculated from a standard curve and reported as nanomoles per milligram of protein (nmol/mg protein), serving as a biochemical marker of oxidative stress.

### 4.8. GSH

GSH concentrations were assessed using Ellman’s colorimetric method [40], which detects reduced GSH through its reaction with 5,5′-dithiobis(2-nitrobenzoic acid) (DTNB). Briefly, proteins in the striatal homogenate were precipitated by adding 10% trichloroacetic acid, followed by centrifugation at 8000× *g* for 10 min. The resulting supernatant was reacted with 1.0 mL of Ellman’s reagent, prepared by dissolving 19.8 mg of DTNB in 100 mL of 1.0% sodium citrate and mixing it with 3 mL of phosphate buffer (pH 8.0). The yellow-colored product formed from the interaction between DTNB and free thiol groups in GSH was measured at 412 nm. GSH levels were quantified against a standard curve and expressed as nanomoles per milligram of tissue (nmol GSH/mg tissue).

### 4.9. SOD Activity

SOD activity was evaluated using the method originally developed by Misra and Fridovich [41], which relies on the enzyme’s ability to inhibit the spontaneous oxidation of epinephrine to adrenochrome under alkaline conditions. For the determination of SOD activity, a reaction mixture was prepared by adding 0.05 mL of the tissue supernatant to 2.0 mL of carbonate buffer, followed by 0.5 mL of ethylenediaminetetraacetic acid (EDTA). The enzymatic reaction was initiated by introducing 0.5 mL of a freshly prepared epinephrine solution (3 × 10^−4^ M). The auto-oxidation of epinephrine to adrenochrome was used as an indirect measure of SOD activity, and the reaction kinetics were monitored spectrophotometrically at 480 nm. Absorbance was recorded at one-minute intervals, with the rate of adrenochrome formation reflecting the inhibitory effect of SOD on superoxide radical generation. A reagent blank lacking the sample was used as a reference. One unit of SOD activity was defined as the quantity of enzyme capable of reducing epinephrine oxidation by 50%. Activity values were normalized to total tissue protein and reported as nanomoles of SOD activity per unit per milligram of tissue (nmol SOD/unit/mg tissue).

### 4.10. CAT Activity

CAT activity was determined following the procedure outlined by Beers and Sizer [36], which measures the decomposition rate of hydrogen peroxide (H_2_O_2_) as catalyzed by the enzyme. The reaction mixture included 2 mL of phosphate buffer (pH 7.0), 0.95 mL of 0.019 M H_2_O_2_, and 0.05 mL of the tissue supernatant, yielding a total volume of 3 mL. The reaction was initiated upon addition of the supernatant, and the decline in absorbance at 240 nm was recorded every 10 s for a total of 60 s. CAT activity was determined by measuring the decomposition rate of H_2_O_2_ at 25 °C and pH 7.0. Enzymatic activity was defined as the quantity of CAT required to decompose 1 mmol of H_2_O_2_ per minute under the assay conditions. Results were standardized and reported as CAT units per milligram of tissue (units CAT/mg tissue), using a calibration curve generated from known H_2_O_2_ concentrations for quantification.

### 4.11. Mitochondrial Function

To isolate mitochondria from rat striatal tissue, a modified differential centrifugation method was employed, based on the protocol described by Moreadith and Fiskum [42]. A 10% tissue homogenate was prepared using an ice-cold Tris-sucrose buffer (0.25 M, pH 7.4) with a glass-Teflon homogenizer maintained at 4 °C. Initial centrifugation at 1000× *g* for 10 min removed nuclear debris. The supernatant was then centrifuged at 10,000× *g* for 20 min to separate cytosolic and mitochondrial fractions. The resulting mitochondrial pellet was washed three times in a mannitol–sucrose–HEPES buffer (pH 7.4) and resuspended in the same buffer for subsequent biochemical assays. Succinate dehydrogenase (SDH) activity, a marker of mitochondrial complex II function, was evaluated using a modified Pennington method [43]. In brief, 0.05 mg of isolated mitochondrial protein was incubated with 50 mM potassium phosphate buffer (pH 7.4), 0.01 M sodium succinate, and 2.5 µg/mL p-iodonitrotetrazolium violet for 10 min. The reaction was halted with 10% trichloroacetic acid (TCA), and the resulting formazan product was extracted using a solvent mixture of ethyl acetate, ethanol, and TCA (5:5:1, *v*/*v*/*w*). Absorbance was measured at 490 nm, and SDH activity was expressed as optical density per milligram of mitochondrial protein. Total ATPase activity was measured based on the release of inorganic phosphate from ATP, following the method of Prasad and Muralidhara [44]. The reaction mixture included 50 µg of cytosolic protein, 0.02 M Tris-HCl buffer (pH 7.4), 100 mM NaCl, 20 mM KCl, and 5 mM MgCl_2_. Samples were incubated at 37°C for 15 min, after which the reaction was terminated with 20% TCA. Following centrifugation at 15,000× *g* for 10 min, the concentration of free inorganic phosphate in the supernatant was measured colorimetrically. Blank samples lacking enzyme were used as negative controls. ATPase activity was reported as micrograms of phosphate released per milligram of protein. Activities of NADH-cytochrome C reductase (complex I–III) and succinate-cytochrome C reductase (complex II–III), which reflect mitochondrial electron transport chain integrity, were quantified using established spectrophotometric protocols [45].

### 4.12. Inflammatory Markers 

The concentrations of neuroinflammatory cytokines—TNF-α, IL-1β, and IL-6—in rat tissue samples were determined using commercially available enzyme-linked immunosorbent assay (ELISA) kits supplied by KRISHGEN BioSystem (Ashley Ct, Whittier, CA, USA). These assays employed a solid-phase sandwich ELISA design (Quantikine format), specifically optimized for accurate quantification of cytokines in rat-derived biological matrices. All procedures were carried out in accordance with the manufacturer’s protocols, which followed a standardized 4.5-h workflow. Cytokine levels were assessed spectrophotometrically using a microplate reader, with concentrations calculated from standard curves generated using serial dilutions of known cytokine standards. Final values were normalized against total protein content and expressed in picograms per milliliter (pg/mL).

### 4.13. Caspase-3 Activity

Caspase-3, also known by aliases such as CPP-32, Apopain, or Yama, is a critical executioner protease involved in the final stages of the apoptotic cascade. Synthesized initially as an inactive zymogen (pro-caspase-3), it is activated via cleavage upon exposure to apoptotic signals. Once activated, caspase-3 cleaves various intracellular substrates, initiating both morphological and biochemical hallmarks of programmed cell death. Quantitative analysis of caspase-3 activity was carried out using a colorimetric detection kit (Cat No. GTX85558, GeneTex Inc., Hsinchu, Taiwan), which employs synthetic peptide substrates specific to caspase-3—typically containing the recognition sequence DEVD and conjugated to the chromogenic reporter p-nitroaniline (pNA). Enzymatic cleavage at the DEVD site releases free pNA, leading to a measurable absorbance increase at 405 nm. Caspase-3 activity in the samples was thus directly proportional to the optical density at this wavelength, with results expressed as nanomoles of pNA released per milligram of total protein (nmol/mg protein).

### 4.14. Protein Quantification 

Protein concentrations in both cytosolic and mitochondrial fractions were determined by the method of Lowry et al. [46], with bovine serum albumin (BSA; Sigma, St. Louis, MO, USA) used as the calibration standard. Absorbance readings were taken at the appropriate wavelength, and protein levels were interpolated from a standard curve constructed with known BSA concentrations.

### 4.15. Statistical Analysis

All experimental data are reported as mean ± standard error of the mean (SEM). Behavioral data were analyzed using two-way repeated measures analysis of variance (ANOVA) to account for both time and treatment effects. Biochemical parameters were evaluated using one-way ANOVA, followed by Tukey’s post hoc test for multiple comparisons. A *p*-value less than 0.05 was considered statistically significant. All statistical analyses were performed using GraphPad Prism software, version 8.3.0 (GraphPad Software Inc., San Diego, CA, USA).

## 5. Conclusions

In summary, our findings provide compelling evidence supporting the neuroprotective effects of GRN in the treatment of OD in a well-established animal model. These protective effects appear to be mediated through multiple mechanisms, including antioxidant activity, preservation of mitochondrial function, inhibition of neuroinflammation, and suppression of apoptotic pathways—potentially via modulation of the Nrf2 signaling pathway. Further investigations are required to determine whether the neuroprotective effects of GRN are mediated through the modulation of the Nrf2 signaling pathway, including assessments of Nrf2 activation and its downstream targets. Given that HPD-induced OD in rats is a widely accepted and translationally relevant model for exploring the pathophysiology of TD, our results provide a promising foundation for future clinical exploration of GRN as a potential adjunctive therapy aimed at mitigating TD-related neurotoxicity. However, further research is necessary to facilitate the clinical translation of these preclinical findings. This should include comprehensive pharmacokinetic and pharmacodynamic evaluations, dose optimization, and rigorous safety assessments to determine GRN’s tolerability and potential adverse effects in human subjects. Such investigations will be critical for establishing the therapeutic viability of GRN in the clinical management of TD.

## Figures and Tables

**Table 1:** The frequencies of VCM were used as the output measurements to assess HPD-induced OD behavioral characteristics.

VCM (Counts/5 min)				
	Day 1	Day 7	Day 14	Day 21
C	1.14 ± 0.69	1.29 ± 0.95	1.29 ± 0.76	1.14 ± 0.69
H	18.86 ± 2.54 ^a^	25.14 ± 2.1 ^a^	35.57 ± 2.51 ^a^	58.86 ± 2.85 ^a^
G1	0.86 ± 0.69	1.24 ± 0.9	11 ± 0.82	1.29 ± 0.76
G3	1.28 ± 0.76	1 ± 0.82	1.15 ± 0.38	1.15 ± 0.69
G10	0.71 ± 0.85	0.89 ± 0.9	1.22 ± 0.48	1.38 ± 0.38
G30	1.37 ± 0.78	0.86 ± 0.9	1.17 ± 0.58	1.23 ± 0.77
G100	1.3 ± 0.79	1.15 ± 0.57	1.37 ± 0.38	1.33 ± 0.59
H + G1	17.23 ± 2.64 ^a^	24.95 ± 3.32 ^a^	33.57 ± 3.43 ^a^	57.96 ± 2.77 ^a^
H + G3	19.12 ± 2.66 ^a^	25.23 ± 2.46 ^a^	35.48 ± 2.95 ^a^	56.81 ± 2.54 ^a^
H + G10	17.47 ± 3.01 ^a^	25.69 ± 2.95 ^a^	34.47 ± 3.44 ^a^	39.14 ± 2.73 ^ab^
H + G30	18.22 ± 3.09 ^a^	24.45 ± 2.17 ^a^	26.86 ± 2.1 ^ab^	29.86 ± 2.61 ^ab^
H + G100	17.93 ± 2.73 ^a^	23.66 ± 2.9 ^a^	24.45 ± 3.05 ^ab^	25.74 ± 2.98 ^ab^
H + M + G10	17.86 ± 3.43 ^a^	23.86 ± 2.61 ^a^	34.14 ± 2.97 ^a^	56.29 ± 2.06 ^a^
H + M + G30	17.14 ± 1.86 ^a^	23.71 ± 2.56 ^a^	33.86 ± 2.48 ^a^	55.14 ± 2.41 ^a^

Each VCM assessment session was conducted over a duration of 5 min. The frequency of these purposeless chewing actions was recorded as an indicator of orofacial dyskinesia. Results are presented as the mean ± standard error of the mean (S.E.M.), with a sample size of eight animals per group (*n* = 8). Statistical comparisons were carried out using a two-way analysis of variance (ANOVA), followed by Tukey’s post hoc test to identify group differences. Statistical significance was defined as follows: “a” *p* < 0.001 relative to the control group (C); “b” *p* < 0.001 relative to the HAL-treated group (H).

**Table 2:** The frequencies of TP were used as the output measurements to assess HPD-induced OD behavioral characteristics.

TP (Counts/5 min)				
	Day 1	Day 7	Day 14	Day 21
C	1.71 ± 0.75	1.85 ± 0.69	1.43 ± 0.79	1.29 ± 0.49
H	7.43 ± 1.62 ^a^	11.29 ± 1.6 ^a^	16.71 ± 1.6 ^a^	26.57 ± 3.1 ^a^
G1	1.85 ± 1.06	1.71 ± 0.76	1.86 ± 0.38	1.57 ± 0.98
G3	2.14 ± 0.38	1.14 ± 0.38	2.28 ± 0.76	1.71 ± 1.11
G10	2.09 ± 0.76	1.89 ± 0.9	1.68 ± 0.68	1.44 ± 0.51
G30	1.72 ± 0.88	1.45 ± 0.78	1.69 ± 0.67	2.23 ± 0.98
G100	2.13 ± 0.89	1.85 ± 0.81	1.97 ± 0.78	1.83 ± 0.79
H + G1	7.23 ± 2.64 ^a^	10.95 ± 2.32 ^a^	15.47 ± 3.56 ^a^	25.96 ± 2.27 ^a^
H + G3	7.46 ± 1.55 ^a^	10.77 ± 2.54 ^a^	15.48 ± 2.97 ^a^	24.81 ± 3.14 ^a^
H + G10	7.11 ± 1.37 ^a^	11.12 ± 2.39 ^a^	14.57 ± 2.37 ^a^	18.57 ± 2.23 ^ab^
H + G30	7.29 ± 1.98 ^a^	10.14 ± 1.95 ^a^	10.86 ± 1.57 ^ab^	13.86 ± 1.95 ^ab^
H + G100	7.07 ± 2.1 ^a^	10.06 ± 1.87 ^a^	8.55 ± 2.11 ^ab^	11.45 ± 2.75 ^ab^
H + M + G10	7.29 ± 1.11 ^a^	11.14 ± 1.68 ^a^	15.71 ± 2.43 ^a^	25.57 ± 3.6 ^a^
H + M + G30	7.14 ± 1.57	10.86 ± 1.57 ^a^	14.86 ± 2.91 ^a^	25.54 ± 3.48 ^a^

Each TP evaluation session was carried out over a 5-min period, during which the number of spontaneous tongue extensions was recorded as a behavioral marker of orofacial dyskinesia. The results are expressed as mean values ± standard error of the mean (S.E.M.), with eight animals per experimental group (*n* = 8). Statistical evaluation was conducted using two-way ANOVA, followed by Tukey’s post hoc test to determine intergroup significance. Differences were considered statistically significant at “a” *p* < 0.001 relative to the control group (C); “b” *p* < 0.001 relative to the HAL-treated group (H).

## Data Availability

The datasets generated and analyzed during the current study are available from the corresponding author upon reasonable request. Please note that these data are not publicly accessible in order to maintain confidentiality and protect participant privacy.
